# Sequencing and characterization of the complete mitochondrial genome of *Thinopyrum obtusiflorum* (DC.) Banfi, 2018 (Poaceae)

**DOI:** 10.1080/23802359.2022.2054378

**Published:** 2022-03-25

**Authors:** Xiaojun Wu, Xigui Hu, Xiangdong Chen, Jinlong Zhang, Cuicui Ren, Lintong Song, Fang Fang, Na Dong, Tiezhu Hu, Zhengang Ru

**Affiliations:** a Center of Wheat Research, Henan Institute of Science and Technology, Xinxiang, China; b Henan Key Laboratory of Hybrid Wheat, Xinxiang, China; c Henan Collaborative Innovation Center of Modern Biological Breeding, Xinxiang, China

**Keywords:** *Thinopyrum obtusiflorum*, mitochondrial genome, Poaceae, phylogenetic analysis

## Abstract

In this study, the mitochondrial genome of *Thinopyrum obtusiflorum* was sequenced, assembled, and annotated. The complete circular mitogenome of *Th. obtusiflorum* is 390,725 bp in length and the overall A + T content of mitogenome is 55.61%. It harbors 33 protein-coding genes (PCGs), 21 transfer RNA genes (tRNAs), six ribosomal RNA genes (rRNAs), and 20 simple sequence repeats (SSRs). Phylogenetic analysis indicates that *Th. obtusiflorum* is a sister to the clade including *Aegilops speltoides*, *Triticum aestivum*, and *Triticum aestivum* cultivar Chinese Yumai in the Triticeae.

*Thinopyrum obtusiflorum* (DC.) Banfi (≡*Thinopyrum ponticum* (Podp.) Barkworth & D.R.Dewey, 1985, 2*n* = 10× =70) is a perennial allodecaploid species in the Triticeae (Poaceae), mainly native to European and Near Asian East (Banfi 2018; Tiryaki et al. 2021). It is known to possess many elite genes for wheat improvement, such as those for wheat leaf rust resistance (Sepsi et al. 2008), stem rust resistance (Mago et al. 2019), stripe rust resistance (Wang et al. 2020), powdery mildew resistance (He et al. 2017), Fusarium head blight (FHB) resistance (Forte et al. 2014), and tolerance to abiotic stress, such as cold, drought and salinity (Friebe et al. 1996; Linc et al. 2012). All of these characters, coupled with its high cross-compatibility with wheat genome, make this species an important donor of elite genes for improving the genetic diversity of cultivated wheat.

The fresh leaves of *Th. obtusiflorum* were collected from the Botanical Garden of Center of Wheat Research, Xinxiang, Henan, China (113°88′E 35°30′N). The voucher specimen was deposited at the Herbarium of Henan Institute of Science and Technology, Xinxiang, China (Zhengang Ru; rzgh58@163.com) under the voucher number XM-W027. Total genomic DNA was extracted using a modified cetyltrimethylammonium bromide (CTAB) method. Sequencing was carried out on the Illumina NovaSeq 6000 platform and PacBio Sequel platform. The *Th. obtusiflorum* mitogenome sequences were assembled with the NovoPlasty v.4.3.1 (Dierckxsens et al. 2017), SPAdes-3.13.0 package (Antipov et al. 2016) and Canu v2.1.1 package (Koren et al. 2017), and then annotated using the online GeSeq tool (Tillich et al. 2017) with default parameters to predict protein-coding genes (PCGs), transfer RNA (tRNA) genes, and ribosomal RNA (rRNA) genes. In addition, the simple sequence repeats (SSRs) were detected in the mitogenome using MISA (MIcroSAtellite Identification Tool) (Beier et al. 2017).

The entire mitogenome of *Th. obtusiflorum* (GenBank accession number: OK120846) is a closed circular double-stranded structure of 390,725 bp in length with base compositions of 27.62% A, 21.96% G, 22.43% C, 27.99% T, exhibiting A + T bias (55.61%). The mitogenome encodes a typical 60 genes, including 33 PCGs, 21 tRNA genes, and six rRNA genes. The cumulative length of the 33 PCGs is 31,404 bp, and the average length of a single-gene is 952 bp, which accounts for 8.04% of the complete genome. The cumulative length of the tRNA genes is 1590 bp, with an average gene length of 75 bp, accounting for 0.41% of the complete genome. The lengths of rRNA genes are 1977 bp (rrn18), 3467 bp (rrn26), and 122 bp (rrn5), which together account for 0.09% of the complete genome. The cumulative length of 20 SSR loci identified is 212 bp which comprises approximately 0.05% of the genome length.

To investigate the evolutionary relationship of *Th. obtusiflorum*, the mitogenome of the other nine species was downloaded from GenBank (https://www.ncbi.nlm.nih.gov/genbank/). Phylogenetic tree was constructed based on functional protein-coding sequences (CDS), first aligned them with the online tool MUSCLE under the default setting (Madeira et al. 2019), and then performed the maximum-likelihood (ML) phylogenetic analysis using PHYML 3.0 (Guindon et al. 2010). The results of phylogenetic analysis supported a monophyletic relationship of *Th. obtusiflorum* to *Aegilops speltoides*, *Triticum aestivum*, and *Triticum aestivum* cultivar Chinese Yumai (Figure 1). This study might be helpful for promoting its utilization of distant hybridization in genetic improvement of wheat.

**Figure 1.**
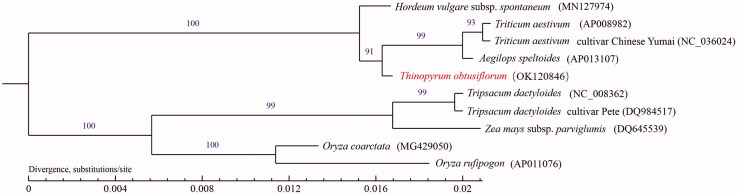
Maximum-likelihood phylogenetic tree based on complete mitogenome of *Thinopyrum obtusiflorum* and other nine species of Poaceae. Numbers above the lines represent ML bootstrap values.

## Authors contributions

Conception and design: Xiaojun Wu, Xigui Hu, Tiezhu Hu, and Zhengang Ru; analysis and interpretation of the data: Xiangdong Chen, Jinlong Zhang, and Cuicui Ren; the drafting of the paper, revising it critically for intellectual content: Xiaojun Wu, Lintong Song, Fang Fang, and Na Dong; all authors have approved the final version of the manuscript and all authors agree to be accountable for all aspects of the work.

## Data Availability

The genome sequence data that support the findings of this study are openly available in GenBank of NCBI at https://www.ncbi.nlm.nih.gov/, reference number OK120846. The associated BioProject, Bio-Sample, and SRA numbers are PRJNA782017, SAMN23314009, and SRP347059, respectively.
